# Medical planning for mass-participation running events: a 3-year review of a half-marathon in Singapore

**DOI:** 10.1186/1471-2458-14-1109

**Published:** 2014-10-27

**Authors:** Clive M Tan, Ian Wern Tan, Wai Leong Kok, Melvin C Lee, Vernon J Lee

**Affiliations:** Headquarters Medical Corps, Singapore Armed Forces, 701 Transit Road, Singapore, 778910 Singapore; Headquarters Army Medical Services, Singapore Armed Forces, 701 Transit Road, Singapore, 778910 Singapore

## Abstract

**Background:**

Systematically planning appropriate medical coverage for mass-participation running events is a challenge that has received relatively little attention in the medical literature, despite its potentially severe consequences. In particular, the literature lacks quantitative information on running events that medical planners can utilize for decisions on medical resource allocation and deployment.

**Methods:**

Using a case-study approach, this study provides a detailed quantitative medical services utilization profile for the Singapore Army Half-Marathon, constructed from participant and casualty data spanning three years and comprising over 80,000 data points. Casualty rates for participants of varying age and sex in different running events were also estimated using a multivariate logistic regression model. Qualitatively, planning processes and practices were described and discussed.

**Results:**

The quantitative profile yielded three main findings. Firstly, the analysis reveals that the gross Medical Usage Rate had remained fairly stable at between 16.9 and 26.0 casualties per 10,000 participants over the three years. Secondly, comparing injury types, musculoskeletal and soft-tissue injuries were the most commonly-presented injuries. Thirdly, more casualties presented at the race end-point as compared to the along the race routes. The regression analysis showed that, of the four modeled variables, the longer event distance (21 km vs. 10 km) had the largest effect on the likelihood that a participant would become a casualty. Conversely, being of an older age, being male, and running in a non-competitive event were each associated with lower casualty risk.

**Conclusions:**

The stable and intuitive casualty patterns detailed in this study provide a strong basis for further quantitative research on the medical aspects of running events, as well as for mass-participation sporting events in general. The qualitative aspects of this report may serve as a useful resource to medical planners for running events.

## Background

Running events such as marathons have seen a rise in interest and participation worldwide [1, 2], and have also grown in popularity in Singapore [3]. In Singapore alone, the number of mass-participation running events has grown from 61 in 2012, to 64 in year 2013. Participants at these events are at risk of injuries and, in the event of certain incidents or accidents, even fatalities [4]. Providing the proper medical support for these events ensures the safety of the participants and the continuity of the events [5, 6].

The Singapore Army Half-Marathon is an annual mass-participation running event held in Singapore, a tropical city-state in South-East Asia, since 1992. It is jointly organized by the Singapore Armed Forces Reservist Association (SAFRA) and the Singapore Army, and is the largest half-marathon race in Singapore, with more than 30,000 runners taking part annually.

The medical support for the Singapore Army Half-Marathon is planned for and supported by the Singapore Armed Forces (SAF) annually. The event’s medical committee is headed by a senior military medical officer. The medical support plan has been built upon and refined year-on-year, with minor adjustments depending on the changes in the running route. To provide useful information for planning the subsequent year’s race, casualty data is meticulously collected and analyzed. Limitations are discussed and medical support plans are improved on year-on-year.

Medical aid stations, termed Medical Posts (MPs), are positioned along the running route and are staffed by doctors and medics. They are supported by a fleet of pre-positioned ambulances at the Ambulance Posts (APs) and medics at designated Casualty Collection Points (CCPs). All medical operations are coordinated by the Command Post, which is equipped with an online electronic medical records system that allows real-time casualty data to be shared across all MPs and the Command Post.

The authors have been involved in planning for medical support for mass gathering events in Singapore. The annual Army Half-Marathon (AHM) was chosen as a case study. Analyzing participant and casualty data from three consecutive years of the event (2010–2012), this study provides descriptive analyses of the profile of participants, casualty rates and injury patterns. Variables affecting the risk of a participant becoming a casualty were estimated using logistic regression analysis. This paper concludes with the implications of these findings on medical support planning for large-scale mass-participation running events, along with practical insights that might be useful for medical planners.

## Methods

Participants registered for the event through an online website. Participants provided data on their age and sex, chose the event distance (21 km and 10 km), type of run (competitive vs. non-competitive), and the presence of any drug allergies; this information was collected and collated electronically. They were also required to fill up a Physical Activity Readiness Questionnaire (PAR-Q). If there were potential health risks picked up in the PAR-Q, the participants were asked to have a doctor certify them fit for running before race registration can be duly completed.

During the course of the race event, casualties attended to by the medical staff at the MPs were triaged by the doctors into light, intermediate or severe categories, based on the Simple Triage and Rapid Treatment (START) triage tool [7]. The time at which the casualties were registered, the time at which they left the MPs, the documentation of the injury and records of the medical management rendered were also captured in the medical records. Data from all medical stations was collated into a single casualty dataset for the event.

The casualty dataset was matched to participant registration data and collated into a single dataset for analysis. The resulting master dataset contained 84,644 observations (participants) spanning three years (2010–2). Individuals who participated in multiple years were treated as separate observations for the purpose of quantitative analysis.

Descriptive analysis of the dataset was conducted for participant demographics, casualty statistics, injury patterns, and casualty presentation patterns. Bivariate logistic regression analyses were conducted using participant’s demographic data, event distance, event category, participant’s race information such as start-time, end-time and duration of run, to estimate participant risk for becoming a casualty (outcome). Next, statistically significant variables were used in multivariate logistic regression to quantify participant risk for becoming a casualty.

The data used for the study was obtained from the Singapore Armed Forces Reservist Association (SAFRA) and the Singapore Armed Forces’ Army Medical Services, with permission from the organization’s Joint Medical Committee. All statistical analyses were performed in STATA 11.0 (Statacorp LP, College Station, Texas, USA).

## Results

**Figure 1:**
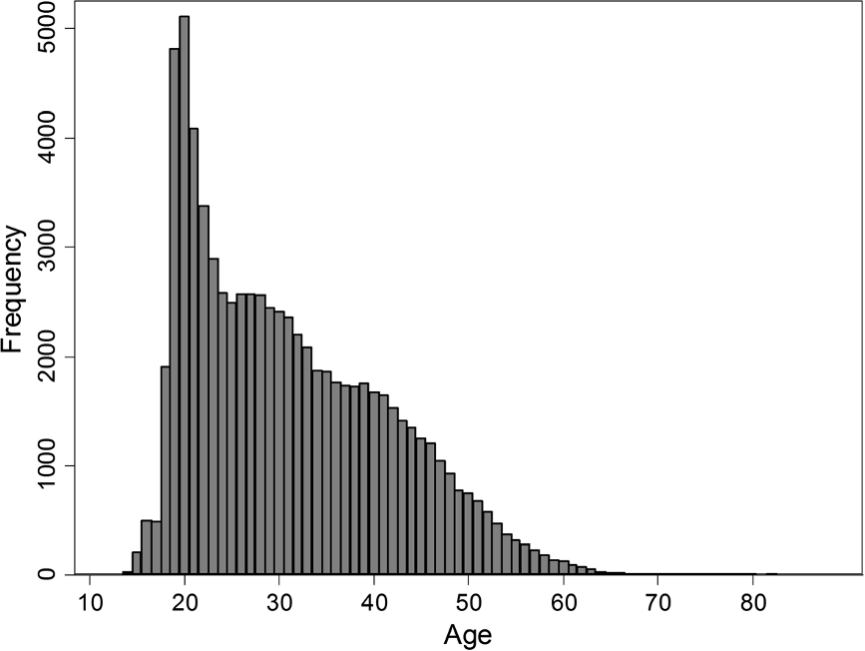
**Age distribution of AHM 2010-2012 participants.**

The number of participants for AHM had increased gradually from 2010 to 2012 (Table 1), possibly reflecting a stronger public interest in running events. 63.9% of participants ran in the competitive category; 58.0% of participants ran in 21 km events and the rest ran in the 10 km event. Males comprised 84.9% of all participants. Figure 1 shows the age distribution of the participants.

**Table 1:** **Casualties by severity and year, including medical usage rate**

	Registered participants	Total casualties	Severe	Intermediate	Light	MUR
2010	26010	66	7	12	47	25.37
2011	34673	90	3	11	76	25.96
2012	38480	65	2	10	53	16.89

**Figure 2:**
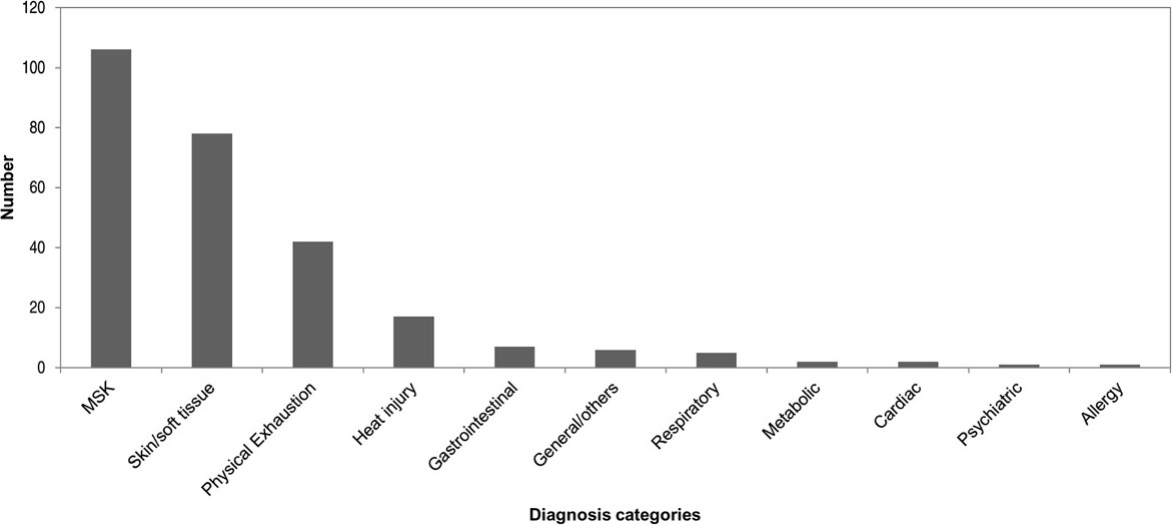
**Medical conditions by category for casualties (AHM 2010-2012).**

**Figure 3:**
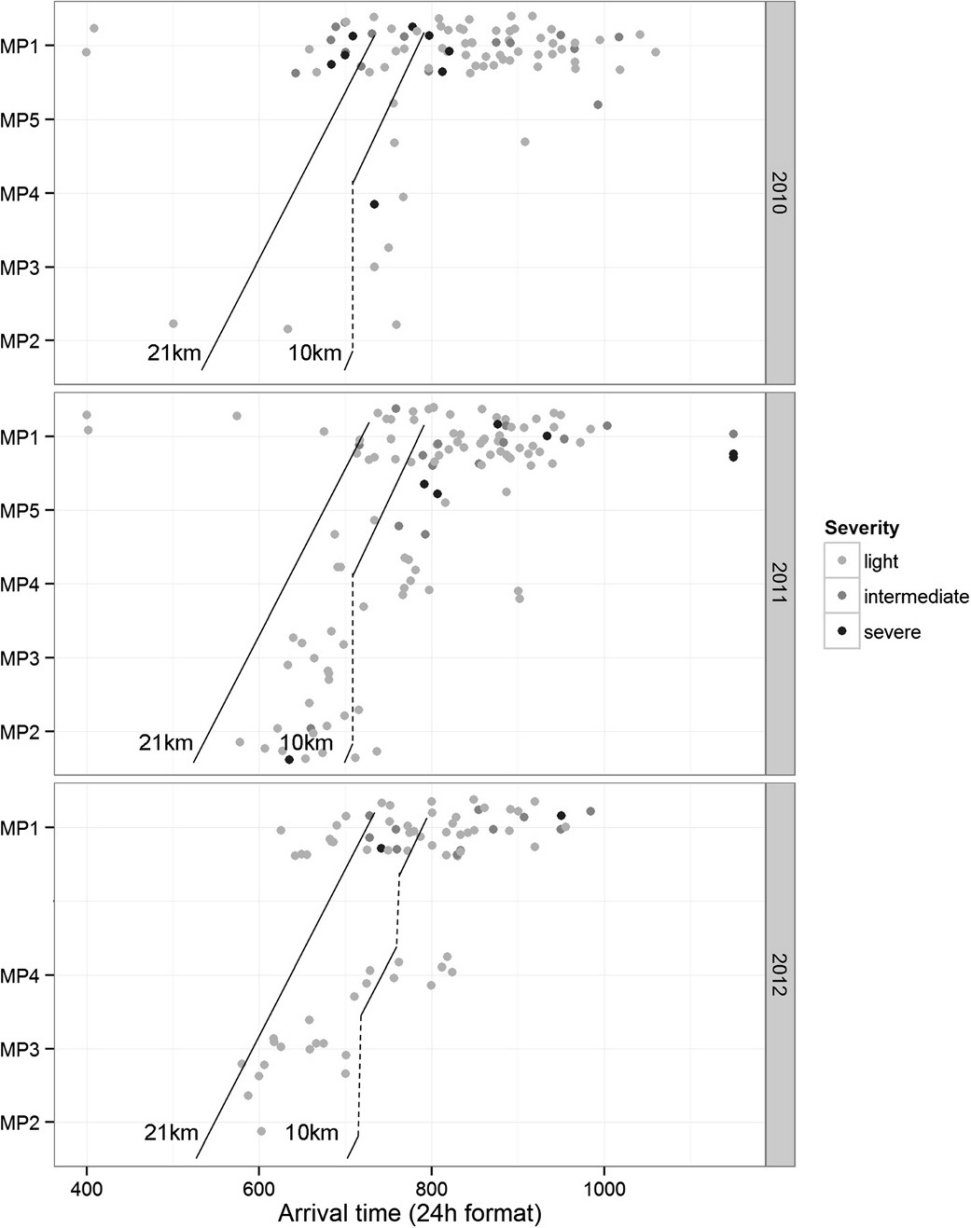
**Spatio-temporal distribution of casualty presentation, by year and severity.**

**Figure 4:**
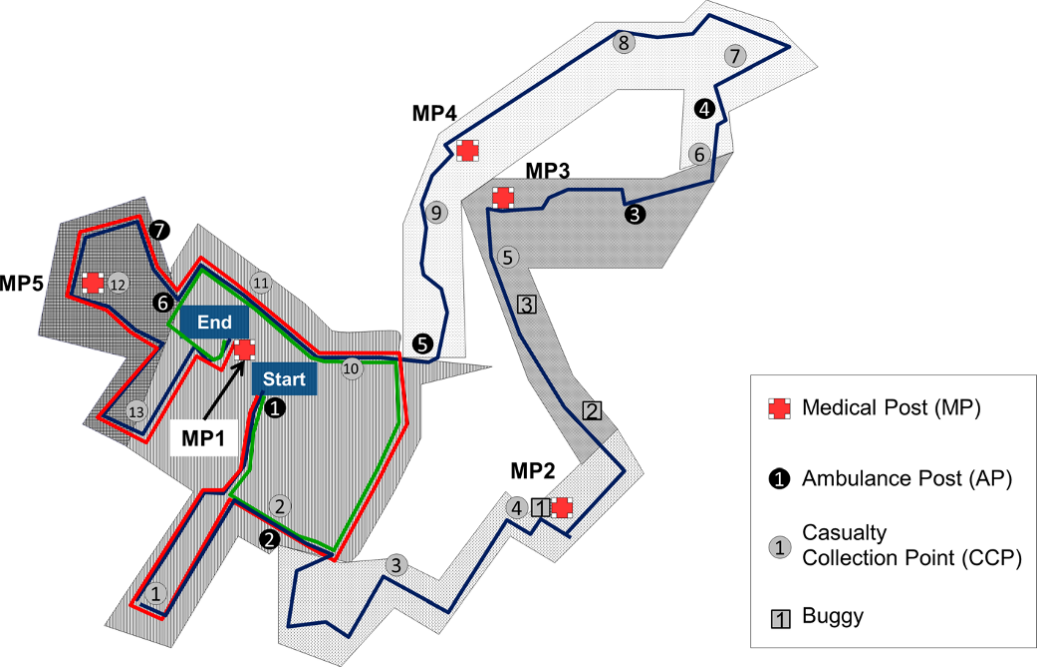
**Schematic diagram of running route and area coverage by each Medical Post, AHM 2011.**

Number of casualties attended to in each year ranged from 65 to 90. Light casualties made up the majority of casualties, followed by intermediate and severe casualties. The MUR, defined as the number of casualties attended to per 10,000 participants, varied within a narrow band from 16 to 26.Casualty records were reviewed and analysed for the medical conditions that the casualties presented with (Figure 2). Musculoskeletal conditions [37.9%] (e.g. muscle cramps, joint sprains) were the most common medical condition seen at these events, closely followed by skin and soft tissue injuries [29.2%] (e.g. skin abrasions, blisters). Physical exhaustion [15.7%] was the third most common diagnosis category, followed by heat injury [6.4%].Figures 3 and 4 jointly illustrate the pattern of casualty presentation. In Figure 3, the casualties’ time and location of presentation were plotted on a scatterplot grid for each of the three years under study, with colors depicting case severity. The black lines depict the running trajectory, with reference to the medical posts, of a participant running at a steady speed of 10 km/h.As illustrated in Figure 3, the 21 km race started at 5.15 am and the 10 km race started at 7.00 am. The highest number of casualties presented at MP1, which was situated at the start/end point. The greatest density of casualties was around 8 am, which was when most runners complete the 21 km and 10 km event. MPs providing support at various intermediate points in the running route see a lower rate of casualties compared to the MP at the start/end point. It should also be noted that a small number of casualties presented before 5.15 am – these were not runners, but were staff from the event organizers.Figure 4 shows the 21 km, 10 km and 5 km running route from the 2011 race event, and includes the geographical location of the MPs as well as the area covered by each MP. The MPs were evenly spaced between each other for efficient allocation of medical resources. Of note, the runners in the 10 km event did not pass by all the available MPs, and this is depicted by the dashed lines in Figure 3.

The results of the multivariate logistic regression for estimating participant risk for becoming a casualty (outcome) is shown in Table 2.

**Table 2:** **Multivariate logistic regression results**

Variable	Beta	Standard error	P-values
*Age*	−0.0161	0.008	*p* ≤ .05
*Male vs Female*	−0.567	0.170	*p* ≤ .001
*Competitive vs Non-competitive*	0.698	0.202	*p* ≤ .001
*10km vs 21km*	1.133	0.182	*p* ≤ .001
Intercept	−6.400	0.303	*p* ≤ .001
N =84,644			

The distance of the event (10 km versus 21 km) had the largest effect in explaining the participant’s risk of becoming a casualty, followed by the competitiveness of the event. Being a female runner also increased the risk of becoming a casualty. The models’ results were used to estimate participant casualty risk in several illustrative runner profiles (Table 3), which are not exhaustive.

**Table 3:** **Regression model estimates of casualty risk for example runner profiles**

Runner profiles	Age	Sex	Competitive?	Distance	Risk
Young male runner (10km, non-competitive)	20	M	No	10km	0.068%
Young male runner (21km, non-competitive)	20	M	No	21km	0.21%
Young male runner (21km, competitive)	20	M	Yes	21km	0.42%
Young female runner (21km, competitive)	20	F	Yes	21km	0.75%
Middle-aged male runner (10km, non-competitive)	40	M	No	10km	0.050%
Middle-aged male runner (10km, non-competitive)	40	M	No	21km	0.15%
Young female runner (10km, competitive)	25	F	Yes	10km	0.22%
Veteran male runner (21km, competitive)	60	M	Yes	21km	0.22%

Table 3 shows that running in the 21 km category instead of the 10 km category is associated with a mean increase in casualty risk of 0.20%. Running in a competitive event compared with a non-competitive event was associated with a mean increase in casualty risk of 0.13%. Compared to male runners, female runners had a 0.11% higher chance to become a casualty. Increasing age was shown to be negatively associated with casualty risk; as shown in Table 3, a 20-year old runner running in a 21 km, competitive event has a 0.42% chance of becoming a casualty, whereas a 60-year old runner running the same event has only 0.22% chance of becoming a casualty. This is a decrease of 0.2% for an age increase of 40 years.

## Discussion

Mass gatherings, defined as an organized event attended by more than 1,000 people at a specific location for a specific period of time, are increasing in numbers in this global and highly interconnected world [8–11]. While this article addresses specifically mass-participation sporting events, mass gathering can also take the form of large conventions, religious gatherings, or secular celebrations, each with their unique considerations and challenges for public health and the local healthcare systems [12–15]. Any event where large number of people gather are at a higher risk for generating casualties and have a greater likelihood of a mass casualty situation [16–18].

Mass-participation sporting events are on the rise across many parts of the world [2, 17, 18]. In Singapore, participation in mass-participation running events, triathlons and other classes of sporting events (e.g. cycling) have been on the rise, and in 2013, there were 64 running events of various magnitudes being organized in Singapore alone. World-wide, the availability of medical support and its level of organization at these events are highly variable. This could be due to many reasons – the presence of sporting legislation that dictates a required level of medical support for such events [19, 20], the level of awareness of safety risk assessment and medical support planning amongst event organizers, the availability and cost of medical resources, etc.

While the number of mass-participation sporting events are on the rise, the casualty data and availability of the events’ medical support plans are often not openly shared. The Singapore Army Half Marathon is an annual event, and the medical support for this event has always been provided by the Singapore Army. The extensive amount of resources made available for the event’s medical support makes it the event with the most comprehensive medical support in Singapore’s running calendar. The yearly post-event review of the casualty data and medical support plans provide deep insights into how the medical support plan can be further optimized. In this case, analysis of the 2011 casualty data showed that one of the medical posts was under-utilised. This led to a decision where the medical committee for the 2012 race decided to just have just 4 medical posts, instead of 5.

The AHM participants’ age and gender profile are not typical of half-marathon events. The event sees a higher proportion of young male runners in their early twenties from the Singapore Army. In contrast, the average age of marathon participants in America was 38.5 years [17], and males comprised 40% of all participants in American half-marathons [2]. Because of the high proportion of young, male participants from the Army, many of whom are “first-timers”, the analysis showed that older runners were at lower risk of becoming casualties. Understanding the local context, this could be because these older runners tend to be more seasoned and experienced runners, who undergo their own build-up training program in preparation for the event. The explanation that older, more seasoned runners are at lower risk of becoming a casualty suggests the effects of self-selection, compared to the young runners who usually join due to organizational or peer pressures. The fact that competitive runners and a longer race distance are associated with a higher casualty risk are in line with conventional wisdom and our knowledge of sports-related morbidity; competitive runners are more likely to push their bodies to the limits and are at an increased risk of developing injuries. Running longer distances (21 km) also places significant additional stress on runners’ bodies compared to shorter distances.

Medical planners will find the analysis of the casualties’ presenting medical conditions (Figure 2) to be a useful guide in the training and equipping of their staff. The Army Half-Marathon’s casualty profile is largely similar to that reported for other running events [20]. A high proportion of casualties were seen for musculoskeletal, skin and soft tissue conditions. Fortunately, despite presenting in large numbers, these conditions are rarely life threatening. From 2010 to 2012, while there were only 2 casualties with cardiac issues (presenting with chest pain), onsite emergency medical services have to be ready for these emergencies. Medical planners have to address their plans for casualties with conditions such as head trauma, heat-related injuries, respiratory and cardiac emergencies, which could be life-threatening if not managed promptly and correctly.Medical support planning for a mass-participation running event is dynamic, over both time and location, as illustrated in Figures 3 and 4. The pattern of casualty presentation illustrated highlights that the workload shifts to different medical posts along the running route with the passage of time. The majority of the casualties will be seen at the race’s end-point, along with most of the severe casualties. The study’s visual representation of the geographical and time distribution of casualties may provide medical planners with insights on how to distribute their medical resources.

Event medical support planning requires dedicated resources, specialized knowledge, and the resources committed should be in accordance with evidence-based international or local standards and guidelines [19, 21]. For the Army Half Marathon events from 2010 to 2012, the Medical Usage Rate (MUR) varied within a narrow band from 16 to 26 per 10,000 participants. While MUR can vary widely for events of a diverse nature [14], this narrow range for the MUR may be expected for an annual event with similar planning parameters for all 3 years [8]. The MURs detailed in this study may hence serve as a useful reference to planners. If planning parameters for future Army Half-Marathons remain largely the same, medical planners can use this estimated casualty rate and the number of participants to broadly estimate the number of casualties they are to expect for that year’s event. As far as the authors are aware, this is the first time such an individual-level analysis of casualty rates has been conducted, and with such a broad range of variables.

To increase the available information on casualty statistics and injury rates, mass-participation running event organizers and medical planners should consider collating and publishing their casualty numbers, statistics and perhaps even making available their medical support plan. With more data available, analysis can yield information that can improve event medical support planning for subsequent years and similar event-types, and in the long-term advance the knowledge in the fields of mass-participation sports event medical support planning and mass gathering medicine.

### Limitations

The use of predictive modeling for casualty rates in this study is of limited precision. Casualty estimates are likely to be sensitive to changes in variables not included in the model, such as weather and temperature. In this study, the temperature and humidity data was collected from the meteorological stations and were largely similar, hence they were not included in the analysis.

The external validity of this study may also be limited. The AHM’s participant demographics are younger with many more male than female participants, unlike most running events. Risk estimates cannot be safely extrapolated to full marathon runners, as it is unknown if risk varies linearly with distance run. Moreover, this study may underestimate the casualty risk for less experienced runners in other marathons. This is because the SAF ensures that even first-time participants receive safe and adequate step-up training to prepare them for the half-marathon. More extensive studies involving different marathons under varying conditions and with a greater variety of participant profiles will be required before we can more accurately estimate casualty risk for similar events.

The analysis also did not take into account the participants from the 5 km running events because the study was intended to focus on mid- and long-distance runs.

In view of these limitations, the estimates generated by these mathematical models should be regarded only as a general guide. The medical support planner should continue to rely on his/her qualitative judgment based on his/her knowledge of best practices and centralize overall responsibility for the event’s medical support.

## Conclusions

Planning medical support for large-scale mass-participation running events is challenging. Understanding the event casualty load over time and location, and associated injury patterns, allow medical planners to better plan and optimize their limited medical resources. The casualty rate for the Singapore Army Half-Marathon appeared fairly constant from 2010 to 2012, the implication being that resource planning can be broadly based on historical data, provided that the participation rate and environment remains fairly constant. Better reporting of casualty statistics and data by event organizers will improve overall understanding of the demands for medical support for similar mass-participation running events.

## Abbreviations

AHM: Army half-marathon
AP: Ambulance post
CCP: Casualty collection point
MP: Medical Post
MUR: Medical usage rate
PAR-Q: Physical activity readiness questionnaire
SAF: Singapore Armed Forces
SAFRA: Singapore Armed Forces Reservist Association
START: Simple triage and rapid treatment.
